# MicroRNA-124 inhibits macrophage cell apoptosis via targeting p38/MAPK signaling pathway in atherosclerosis development

**DOI:** 10.18632/aging.103387

**Published:** 2020-06-30

**Authors:** Xue Liang, Lijun Wang, Manman Wang, Zhaohong Liu, Xing Liu, Baoshuai Zhang, Enzhao Liu, Guangping Li

**Affiliations:** 1 Tianjin Key Laboratory of Ionic-Molecular Function of Cardiovascular Disease, Department of Cardiology, Tianjin Institute of Cardiology, The Second Hospital of Tianjin Medical University, Tianjin 300211, China; 2 Department of Scientific Research, The Second Hospital of Tianjin Medical University, Tianjin 300211, China

**Keywords:** atherosclerosis, p38, miR-124, macrophage, inflammatory response, apoptosis

## Abstract

The objective of this study is to characterize the function of microRNA (miR)-124 in the process of coronary artery disease (CAD). Eighty patients, including 40 CAD patients and 40 non-CAD control patients were enrolled in this study. Atherosclerosis model was established *in vivo* in *ApoE*-/- mice and *in vitro* in RAW264.7 cells. Expression of miR-124 and p38 in patients, animal models and cell models were measured by qRT-PCR, western blot and immunohistochemistry assay. Overexpression or suppression of miR-124 was introduced *in vitro* and *in vivo* and the expression levels of p38, miR-124, pro- and anti-inflammatory cytokines, and pro- and anti-apoptotic factors were examined. Results showed that miR-124 was decreased, while p38 was increased in CAD patients and atherosclerosis models compared with control group. MiR-124 could target p38 by binding its 3’ untranslated region and negatively regulated the protein expression of p38. Overexpression of miR-124 increased the expression of anti-inflammatory cytokines, reduced the expression of pro- inflammatory cytokines, and inhibited macrophage apoptosis. MiR-124 overexpression may be a promising treatment for atherosclerosis and CAD via inhibiting p38.

## INTRODUCTION

Coronary artery disease (CAD) is the one of the leading cause of morbidities and mortalities in elderly people worldwide [1]. It is mainly caused by atherosclerosis which is a life-long process of plaque development within the artery walls, involving several comorbid events including lipid peroxidation and inflammation [2]. The pathogenesis of atherosclerosis is complex and multifactorial, both genetic and environmental risk factors are responsible for this process [3]. Besides, air pollution and fine particulate matter have been recently proved to accelerate atherosclerosis [4, 5].

Atherosclerosis initiates an inflammatory response to the damaged endothelium. Inflammatory mediators rapidly recruit circulating monocytes into the artery wall with platelets arriving and adhering to the damaged area. The recruited monocytes differentiate into macrophages and take up oxidized low-density lipoprotein (ox-LDL), slowly turning into large foam cells that serve as the hallmark of an early step in atherosclerotic plaque formation [6, 7]. Foam cells produce inflammatory factors and macrophage retention factors that further propagate the inflammatory response [6, 7]. It has been gradually accepted that inflammation is involved in all stages of atherosclerotic plaque development [8].

MAPKs includes a wide variety of extracellular signals to cellular responses, including conventional MAPKs such as ERK1 and ERK2, JNKs, p38s, and atypical MAPKs [9]. The p38 MAPKs play a significant role in a variety of inflammatory diseases, including rheumatoid arthritis, atherosclerosis, cardiovascular disease, and inflammatory bowel disease [10–14]. The p38 MAPKs proteins are key therapeutic targets for inflammatory diseases [15]. Four p38 MAPK isoforms have been identified in mammalian, including p38 MAPK α, p38 MAPK β, p38 MAPKγ, and p38 MAPK δ, and among them, p38α is the best characterized and major p38 isoform involved in inflammatory responses [16]. In a clinical trial of a drug used to attenuate the vascular injury-induced inflammatory response, p38 MAPK inhibitor was found to effectively inhibit the expression of the inflammatory marker, high-sensitivity C-reactive protein, after coronary vascular injury [14]. Therefore, inhibition of p38 MAPK might be useful for treating CAD.

In recent years, microRNAs (miRNAs) attracted more attention because of their roles in suppressing protein-coding genes and involving in cellular processes and disease development [17]. For example, a liposome-encapsulated mimic of tumor suppressor miR-34a is designed for treating cancer, and has reached phase I clinical trials [18]. A short oligonucleotide with locked nucleic acid targeting miR-122 for treating hepatitis is under its phase II clinical trials [19]. Therefore, miR-based agents that inhibit p38α activity may serve as the potential treatments for atherosclerosis. In this study, miR-124 was predicted to target p38α using *in silico* methods. Both *in vivo* and *in vitro* experiments confirmed that miR-124 inhibited inflammatory responses and suppressed the expression of p38. MiR-124 overexpression might be a novel therapeutic approach for atherosclerosis by targeting p38.

## RESULTS

### Clinical characters of participants

The clinical features of 80 participants recruited in this study were shown in Table 1. The ratios of participants with a history of cigarette smoking, the LDL level, C-reactive protein level and left ventricular ejection fraction in CAD group were remarkably higher than that in control group (*P* = 0.044, *P* = 0.017, *P* = 0.016 and *P* = 0.002, respectively). Besides, the levels of IL-6 and IL-10 were significantly increased in CAD group in comparison with those in control group (*P* < 0.001). There was no statistically significant difference between the two groups on other parameters, including age, gender distribution, BMI, and blood lipid levels (*P* > 0.05).

**Table 1:** Clinical characters of 80 participants recruited in this study.

	**CAD (n=40)**	**Control (n=40)**	**P value**
Age (year)	60.70 ± 6.42	59.20±5.98	0.283
BMI (kg/m^2^)	25.05±2.47	25.92±2.31	0.107
Smoking (%)	24(60.0%)	15(37.5%)	0.044
HBP (%)	24(60.0%)	19(47.5%)	0.262
DM (%)	16(40.0%)	10(25.0%)	0.152
PLT (×10^9^/L)	197.23±35.13	210.28±30.00	0.078
Cr (umol/L)	72.98±12.66	72.95±12.13	0.993
FBG (mmol/L)	6.22±3.38	5.71±1.28	0.375
ALT (U/L)	20.17±9.17	20.88±7.97	0.711
AST (U/L)	18.92±8.11	17.24±4.12	0.246
TC (mmol/L)	4.18±1.02	4.46±0.79	0.170
TG (mmol/L)	1.81±1.56	1.70±1.0	0.705
HDL (mmol/L)	0.98±0.18	1.03±0.17	0.232
LDL (mmol/L)	2.81±0.69	2.48±0.52	0.017
CRP (mg/L)	4.45±4.63	2.39±2.48	0.016
LVEF (%)	59.30±7.52	63.75±3.92	0.002
IL-6 (pg/ml)	152±47	59±29	< 0.001
IL-10 (pg/ml)	227±59	147±20	< 0.001

CAD, coronary artery disease; BMI, body mass index; HBP, high blood pressure; DM, diabetic mellitus; PLT, platelet; Cr, Creatinine; FBG, fasting blood glucose; ALT, alanine transaminase; AST, aspartate aminotransferase; TC, total cholesterol; TG, triglyceride; HDL, high density lipoprotein cholesterol; LDL, low density lipoprotein cholesterol; CRP, C-reactive protein; LVEF, left ventricular ejection fraction; IL, interleukin.

### MiR-124 and p38 might be involved in the development of atherosclerosis

**Figure 1:**
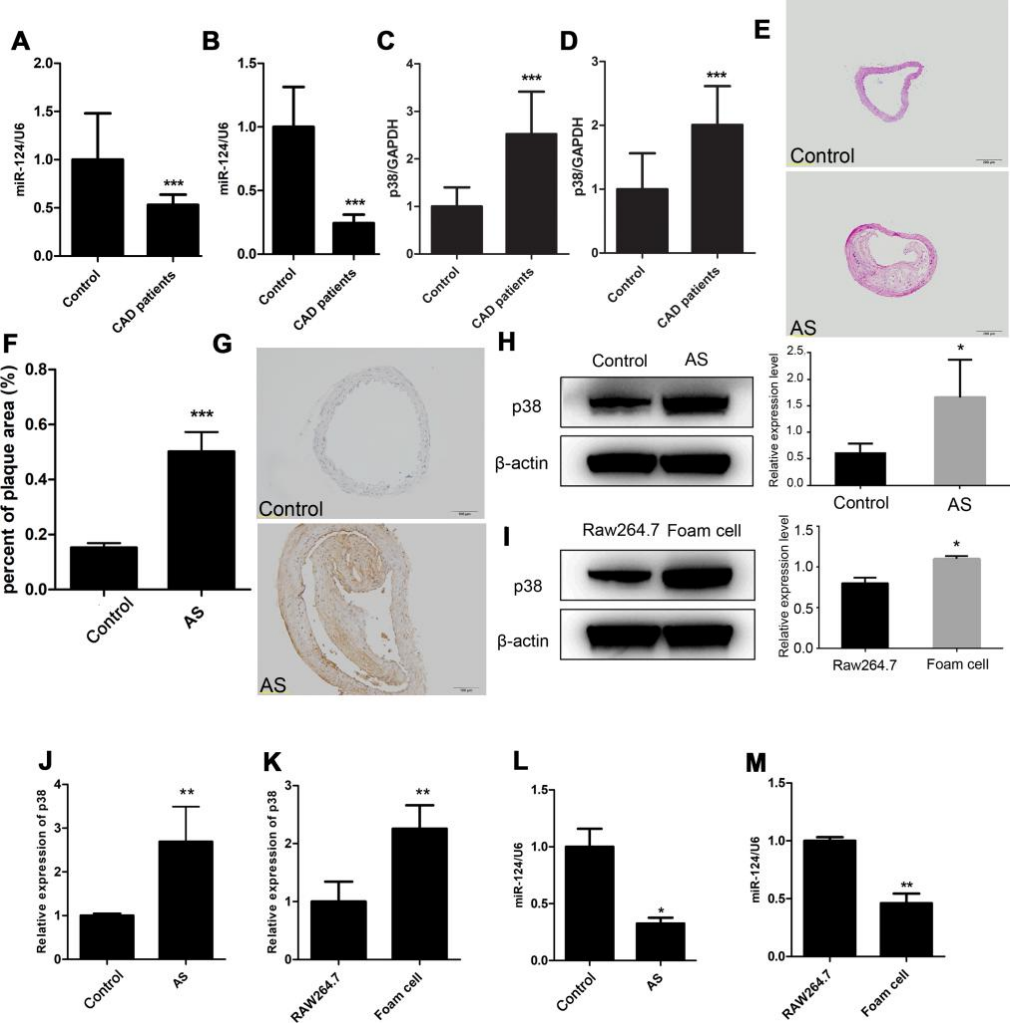
**Expression of miR-124 and p38 in coronary artery disease (CAD).** (**A**) Comparison of miR-124 expression in blood plasma between patients without CAD (n=40) and patients with CAD (n=40). (**B**) Comparison of miR-124 expression in peripheral blood mononuclear cells between patients without CAD (n=40) and patients with CAD (n=40). (**C**) Comparison of p38 expression in blood plasma between patients without CAD (n=40) and patients with CAD (n=40). (**D**) Comparison of p38 expression in peripheral blood mononuclear cells between patients without CAD (n=40) and patients with CAD (n=40). (**E**) Hematoxylin and eosin staining of aorta specimens from atherosclerosis mice and control mice. Bar = 200 μm. (**F**) Quantitative analysis of the percentage of plaque area in control mice and atherosclerosis model mice. (**G**) Immunohistochemistry analysis of p38 protein level in aorta of atherosclerosis mice and control mice. Bar = 100 μm. (**H**) Western blot analysis of p38 protein level in aorta of atherosclerosis mice and control mice. (**I**) Western blot analysis of p38 protein level in RAW264.7 cells and foam cells. The foam cells were induced by treating with oxidized low-density lipoprotein (ox-LDL) for 48 h. (**J**) qRT-PCR analysis of p38 mRNA expression in atherosclerosis mice and the control mice. (**K**) qRT-PCR analysis of p38 expression in RAW264.7 cells and foam cells. (**L**) Stem-loop qRT-PCR analysis of miR-124 expression in atherosclerosis mice and the control mice. (**M**) Stem-loop qRT-PCR analysis of miR-124 expression in RAW264.7 cells and foam cells. β-actin was used as an internal reference in Western blot. GAPDH was used as an internal reference in qRT-PCR assay. U6 was used as an internal reference in stem-loop qRT-PCR. *, *P* <0.05, ** *P* < 0.01 and *** *P* < 0.001 compared with the control (or RAW264.7) group.

The expression of miR-124 and p38 in blood plasma from CAD group and control group were detected by qRT-PCR. Compared with those in control group, the miR-124 expression levels in CAD group were significantly decreased in blood plasma and peripheral blood mononuclear cells (*P* < 0.05, Figure 1A and 1B). On the contrary, the expression levels of p38 in blood plasma and peripheral blood mononuclear cells were significantly increased in CAD group compared with those in control group (P < 0.05, Figure 1C and 1D).

**Figure 2:**
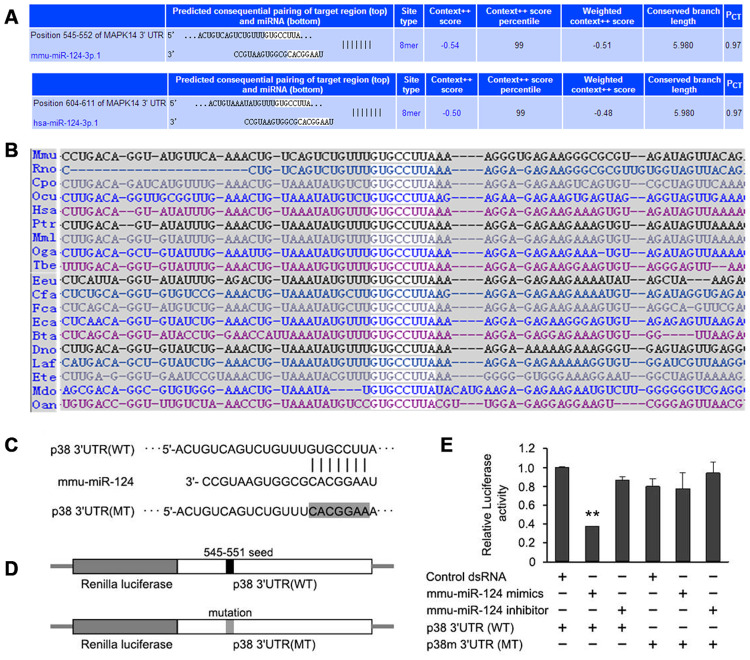
**p38 is predicted to be a putative target of miR-124 by TargetScan.** (**A**) Pairing of hsa-miR-124 and mmu-miR-124 with the 3’-untranslated region (UTR) of *p38* gene from human and mouse, respectively. (**B**) A conserved 8-mer target seed region (gray) of the miR-124 binding site was found within the 3’-UTR of p38 among different mammalian species. (**C**) The original miR-124 binding site and mutated binding site in 3’-UTR of p38. The seven bases of binding sequence (GUGCCUU) for miR-124 on p38 mRNA were mutated to CACGGAA. (**D**) Two types of recombinant luciferase reporter vectors were constructed by replacing the original luciferase mRNA 3’-UTR with the wild-type or mutated 3’-UTR of p38. (**E**) The relative luciferase activity in foam cells detected by dual luciferase reporter assay. NC, negative control of miR-124 mimics; iNC, negative control of miR-124 inhibitor; 3’UTR, 3’-untranslated region; WT, wild type; MT, mutant. ** *P* < 0.01 compared with control group.

The aorta from the ApoE-/- C57B/L6J mice and the littermate control sacrificed after 20 weeks of atherogenic diet was sectioned and stained with H&E. As shown in Figure 1E and 1F, extensive atherosclerotic lesions were visible beneath the valve leaflet in the ApoE-/- C57B/L6J mice compared to the control, suggesting atherosclerosis was successfully induced in ApoE-/- C57B/L6J mice. Further, IHC result showed that the p38 protein level was increased in ApoE-/- C57B/L6J mice compared with that in control mice, and western blot assay confirmed this result (Figure 1G and 1H). qRT-PCR assay also detected an increase in p38 mRNA level in the thoracic aorta tissues of ApoE-/- C57B/L6J group (Figure 2J). Stem-loop qRT-PCR detected that the miR-124 expression level in the thoracic aorta tissues was downregulated in ApoE-/- C57B/L6J mice compared with that in the control mice (Figure 1L), suggesting that the expression of miR-124 was negatively correlated with the p38 level during atherosclerosis development.

After 24 h treatment with ox-LDL, RAW264.7 macrophage cells were induced into foam cells (Supplementary Figure 1). qRT-PCR and western blot showed that miR-124 was significantly decreased while p38 was significantly increased in foam cells compared with the control (Figure 1I, 1K and 1M).

### p38 was predicted to be a putative target of miR-124

*In silico* analysis identified an 8 bp motif in the 3’-UTR of p38 that were complementary to the seed region of miR-124 (Figure 2A). The binding site of miR-124 in human p38 3’-UTR was located between the nucleotide position 604 and 611 nt, and the binding site in mouse p38 3’-UTR was between the nucleotide position 545 and 552 nt. The 8 bp seed region of miR-124 binding site was highly conserved among different mammalian species (Figure 2B), suggesting miR-124 may functionally interact with p38.

In order to clarify whether miR-124 exerted inhibition function by directly binding to the 3’-UTR of p38, the 3’-UTR of luciferase reporter gene in the psiCHECK^TM^-2 vector was replaced with the wild-type 3’-UTR or mutated 3’-UTR of p38. In the mutated 3’-UTR of p38, seven bases (GUGCCUU) in the original miR-124 binding site were changed to CACGGAA (Figure 2C and 2D). The luciferase activity was significantly inhibited in cells transfected with wild-type p38 3’-UTR, while it was not obviously changed in cells transfected with mutated p38 3’-UTR (Figure 2E). This result implied that miR-124 might target the 3’-UTR of p38 to inhibit its expression.

### MiR-124 involved in atherosclerosis development by targeting p38/MAPK signaling pathway

**Figure 3:**
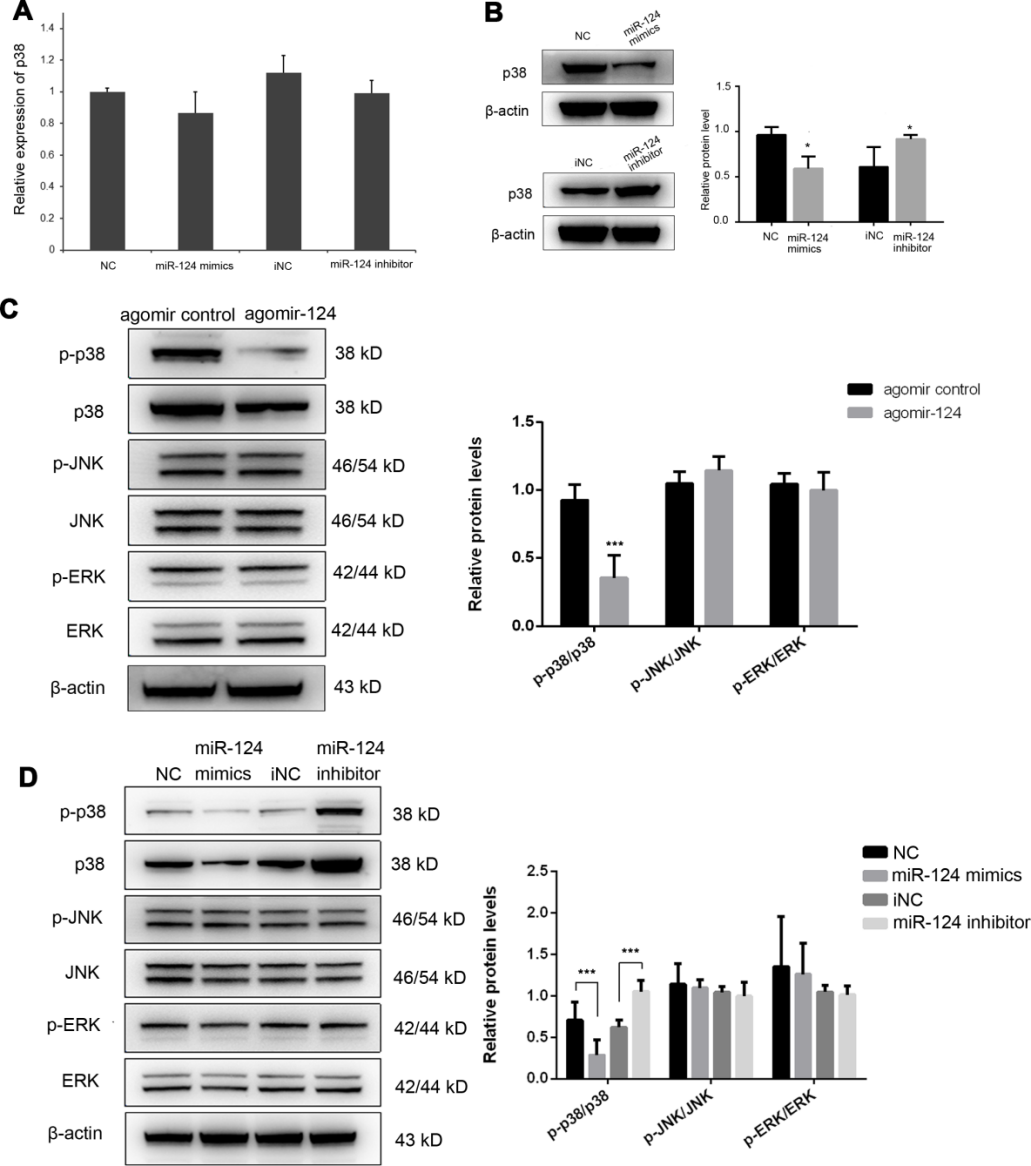
**MiR-124 inhibits the mRNA translation of p38 by directly binding to its 3’-untranslated region (UTR).** (**A**) MiR-124 mimics and inhibitor had no obvious effect on the mRNA expression of p38 in foam cells. (**B**) MiR-124 mimics and inhibitor had reverse effects on the protein expression of p38 in foam cells. MiR-124 mimics reduced while miR-124 inhibitor elevated the protein level of p38 as compared to the controls. (**C**) Western blot analysis of MAPK signaling proteins in aorta of atherosclerosis mice and control mice. (**D**) Western blot analysis of MAPK signaling proteins in RAW264.7 cells transfected with miR-124 mimics or miR-124 inhibitor. GAPDH was used as an internal reference in qRT-PCR assay. β-actin was used as an internal reference in Western blot. *, *P* < 0.05, ***, *P* < 0.001 compared with control group.

The previous study has demonstrated that miR-124 suppressed experimental autoimmune encephalomyelitis by deactivating macrophages [20]. We speculated that it may also function as a modulator of monocyte and macrophage activation in atherosclerosis. Therefore, we further investigated the effect of miR-124 on the expression of p38. The miR-124 expression was greatly increased after transfection of miR-124 mimics, while it was significantly decreased in miR-124 inhibitor group (P < 0.05, Supplementary Figure 2A). The results showed that the transfection of miR-124 mimics or miR-124 inhibitor had no obvious effect on the transcriptional level of p38 (Figure 3A), while transfection of miR-124 mimics significantly decreased the protein level of p38 in RAW264.7 cells as compared with the NC (Figure 3B). On the contrary, transfection of miR-124 inhibitor markedly increased the protein level of p38 in RAW264.7 cells as compared with the NC (Figure 3B). These findings demonstrated that miR-124 might modulate p38 expression by inhibiting its mRNA translation.

Further, the protein expression levels of p-p38, p38, p-JNK, JNK, p-ERK and ERK were detected by western blot *in vivo* and *in vitro*. Administration of agomiR-124 significantly increased the expression of miR-124 in vivo (P < 0.05, Supplementary Figure 2B). *In vivo* results showed that only p-p38/p38 was significantly decreased by agomir-124 (*P* < 0.05), while p-JNK/JNK and p-ERK/ERK were not significantly changed in agomir-124 group (*P* > 0.05; Figure 3C). Similar, in *in vitro* experiments, p-p38/p38 was significantly reduced in miR-124 mimics group while it was significantly increased in miR-124 inhibitor group, compared with those in NC group (*P* < 0.05). However, there was no significant difference on p-JNK/JNK and p-ERK/ERK (*P* > 0.05). These results suggested that miR-124 only target p38 signaling pathway but not JNK or ERK signaling pathways (Figure 3D).

### MiR-124 regulated the expression of pro- and anti-inflammatory cytokines during atherosclerosis development

**Figure 4:**
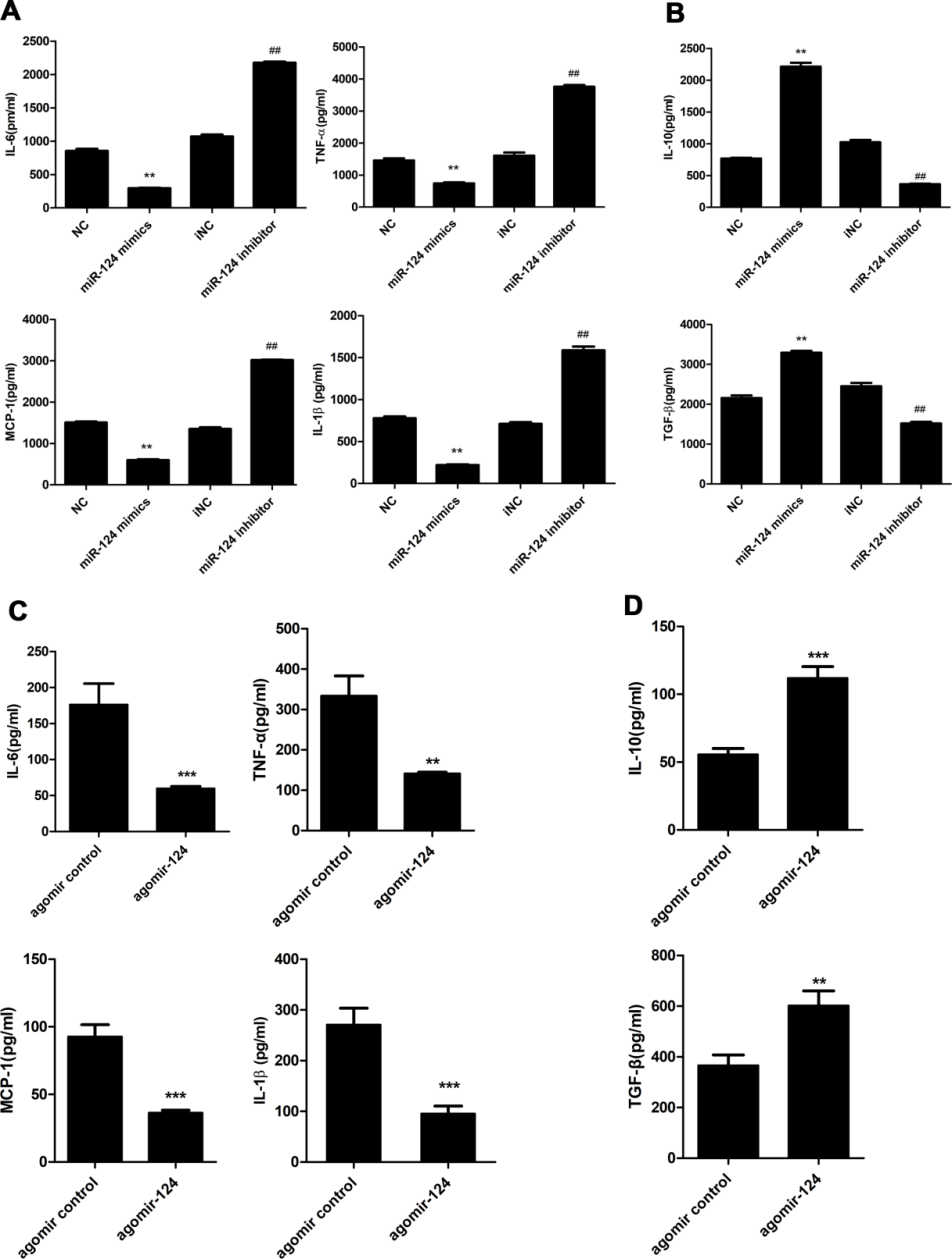
**Effects of miR-124 on pro-inflammatory cytokines and anti-inflammatory cytokines *in vitro* and *in vivo*.** (**A**) ELISA assay analysis of the protein levels of pro-inflammatory cytokines in RAW264.7 macrophage cells. (**B**) ELISA assay analysis of the protein levels of anti-inflammatory cytokines in RAW264.7 macrophage cells. RAW264.7 cells were transfected with the miR-124 mimics, inhibitor or negative control, then treated with ox-LDL. (**C**) ELISA analysis of the protein levels of pro-inflammatory cytokines in blood serum of atherosclerosis model mice treated with agomir control or agomir-124. (**D**) ELISA analysis of the protein levels of anti-inflammatory cytokines. NC, negative control; iNC, negative control of miR-124 inhibitor. **, *P* <0.01, ***, *P* <0.001 compared with control group. ## *P* < 0.01 compared with iNC group.

ELISA was performed to examine the levels of pro-inflammatory cytokines, including IL-1β, IL-6, MCP-1, TNF-α and anti-inflammatory cytokines, including IL-10 and TGF-β in the supernatant of foam cell cultures. The results showed that miR-124 mimics markedly suppressed the expression of IL-6, TNF-α, MCP-1 and IL-1β compared with the NC group, while sharply raised the expression of IL-10 and TGF-β. By contrast, miR-124 inhibitor enhanced the protein levels of IL-6, TNF-α, but attenuated the protein levels of IL-10 and TGF-β in foam cells (Figure 4A and 4B).

As shown in Table 2, agomir-124 treatment did not change the body weight of atherosclerosis mice and the levels of triglyceride, high density lipoprotein cholesterol, and glucose in blood plasma in atherosclerosis mice. However, agomir-124 treatment could significantly decrease the levels of total cholesterol (P < 0.05) and low density lipoprotein cholesterol (*P* < 0.01), indicating that miR-124 might relieve the lipid profile levels of atherosclerosis mice. Meanwhile, ELISA analyses were performed on the blood serum samples from atherosclerosis model mice. As shown in Figure 4C and 4D, agomir-124 significantly suppressed the protein levels of IL-6, TNF-α, MCP-1 and IL-1β, while promoted the protein expression levels of IL-10, TGF-β *in vivo*.

**Table 2:** Body weight and glucose and lipid profile levels of atherosclerosis mice treated with agomir-124 or agomir control.

	**Control (n=10)**	**AS (n=10)**	**agomir control(n=10)**	**agomir-124 (n=10)**
**Body weight (g)**	26.53+1.56	27.89±2.25	29.22±2.95	28.32±0.66
**TG (mmol/L)**	1.09±0.22	5.58±1.81***	4.85±1.96***	3.83±1.31**
**TC (mmol/L)**	3.44±0.48	17.06±4.22***	16.35±3.73***	11.59±2.09***^#^
**HDL (mmol/L)**	1.11±0.18	3.52±0.63***	3.61±0.84***	3.96±0.13***
**LDL (mmol/L)**	1.63±0.59	12.39±3.23***	11.77±2.83***	7.25+1.35**^##&^
**GLU**	6.87 ± 1.31	7.64 ± 0.85	6.72±0.78	5.96±0.86

AS, atherosclerosis; TG, triglyceride; TC, total cholesterol; HDL, high density lipoprotein cholesterol; LDL, low density lipoprotein cholesterol; GLU, blood glucose. **, *** represents *P* < 0.01 or *P* < 0.001 compared with control group; #, ## represents P < 0.05 or P < 0.01 compared with AS group; & represents P < 0.05 compared with agomir control group.

**Figure 5:**
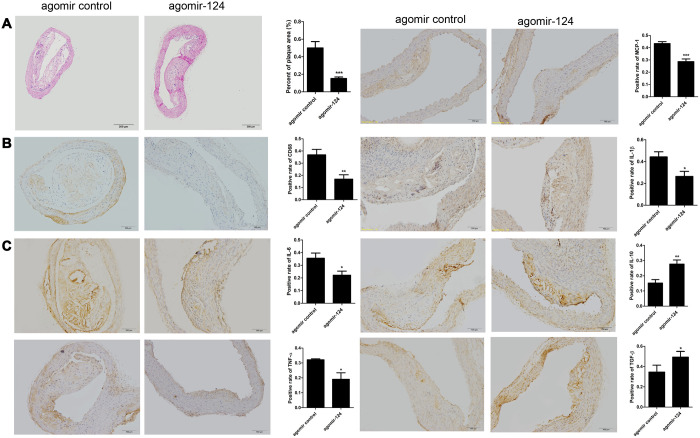
**MiR-124 agomir inhibited CD68, pro-inflammatory cytokines and promoted anti-inflammatory cytokines in the thoracic aorta of atherosclerosis model mice.** (**A**) Hematoxylin and eosin staining of thoracic aorta specimens from atherosclerosis model mice treated with agomir control or agomir-124. Bar = 200 μm. (**B**) Immunohistochemistry analysis of the protein levels of CD68. Bar = 100 μm. (**C**) Immunohistochemistry analysis of the pro-inflammatory cytokines and anti-inflammatory cytokines in the thoracic aorta of atherosclerosis model mice treated with agomir control or agomir-124. Bar = 100 μm. *, *P* <0.01, **, *P* <0.01, ***, *P* <0.001 compared with control group.

The thoracic aorta specimens were collected from atherosclerosis model mice and subjected to H&E staining and IHC assays. H&E staining showed that the intravenous injection of agomir-124 significantly reduced aortic atherosclerotic lesion area in atherosclerosis model compared with the control (*P* < 0.001; Figure 5A). The IHC analysis showed that agomir-124 treatment significantly decreased the staining area of macrophage marker CD68 (*P* < 0.01; Figure 5B) and pro-inflammatory cytokines IL-6, TNF-α, MCP-1, IL-1β, respectively and promoted the staining area of anti-inflammatory cytokine IL-10 and TGF-β, respectively, compared with those in controls (Figure 5C), implying that miR-124 inhibited inflammatory responses during atherosclerosis development.

### MiR-124 could inhibit the apoptosis of atherosclerosis in vivo and in vitro

**Figure 6:**
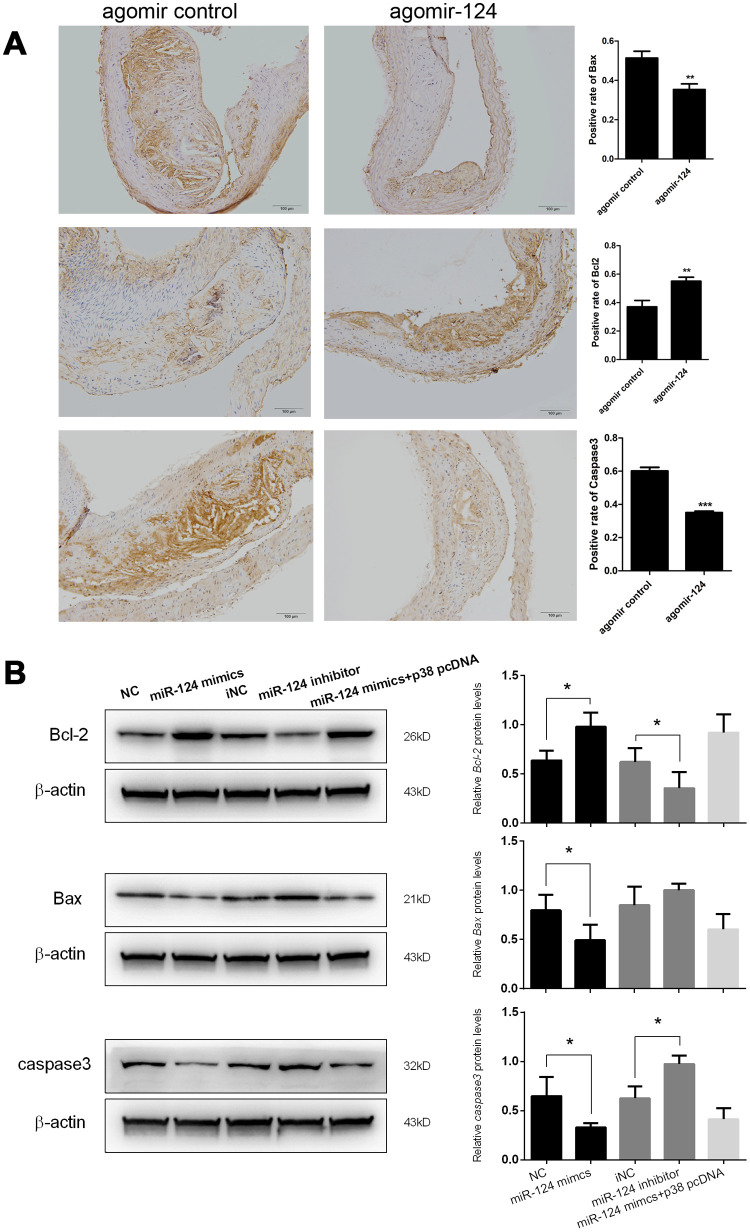
**miR-124 suppressed the expression of pro-apoptotic factors but promoted the expression of anti-apoptotic factors.** (**A**) Immunohistochemistry analysis of the pro-apoptotic factors and anti-apoptotic factors. Bar = 100 μm. (**B**) Western blot analysis of the protein levels of the pro-apoptotic factors and anti-apoptotic factors in RAW264.7 cells transfected with miR-124 mimics, inhibitor, negative control or miR-124 mimics + p38 pcDNA, then treated with ox-LDL. NC, negative control of miR-124 mimics; iNC, negative control of miR-124 inhibitor. β-actin was used as an internal reference in Western blot. *, *P* < 0.05, **, *P* < 0.01, ***, *P* < 0.001 compared with control group.

In addition, the expression levels of pro-apoptotic factors Bax and caspase 3 and anti-apoptotic factor Bcl-2 in atherosclerosis model mice were also examined using IHC assays. The results showed that agomir-124 administration could significantly reduce the staining area of Bax and caspase 3, whereas significantly increase the staining area of Bcl-2 (Figure 6A). This result suggested that miR-124 could prevent cell apoptosis in addition to its anti-inflammatory roles during atherosclerosis development.

At the advanced stage of atherosclerotic plaque development, lesional macrophage apoptosis is associated with plaque necrosis which is a key factor in plaque disruption and acute thrombosis [21, 22]. In view of the function of miR-124 on cell apoptosis during atherosclerosis development *in vivo*, we further investigated the effect of miR-124 on macrophage apoptosis. Western blot showed that Bcl-2 protein expression in foam cells transfected with miR-124 mimics was significantly enhanced, while the Bax and caspase 3 protein levels were greatly reduced compared with the corresponding controls. MiR-124 inhibitor treatment showed a reverse effect on the protein levels of Bcl-2, Bax, and caspase 3 in foam cells. However, there was no significant difference between NC group and the group transfected with miR-124 mimics and p38 pcDNA (Figure 6B).

**Figure 7:**
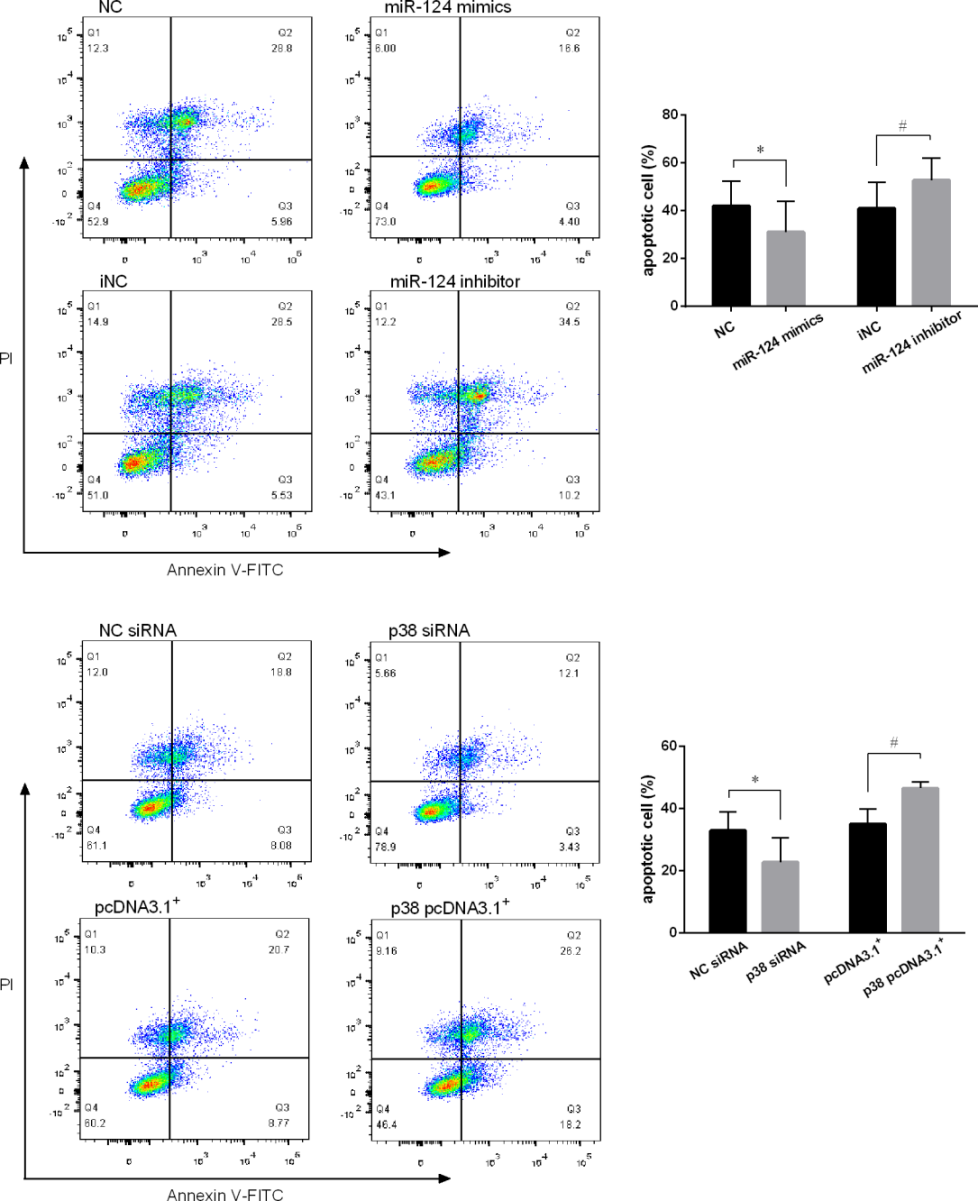
**MiR-124 inhibited the apoptosis and p38 promoted the apoptosis of RAW264.7 cells.** (**A**) Annexin V/PI staining was performed on RAW264.7 cells transfected with the miR-124 mimics, inhibitor or negative control, then treated with ox-LDL for 48 h. After that, the fluorescence was measured using flow cytometry. (**B**) Annexin V/PI staining was performed on RAW264.7 cells transfected with p38 siRNA, p38 pcDNA 3.1. *, # *P* < 0.05.

Annexin V/PI staining was performed on the foam cells and the fluorescence was measured using flow cytometry. As expected, miR-124 mimics decreased the number of apoptotic cells and late apoptotic cells in Q1 and Q2 region, respectively, while increased the number of viable cells in Q3 comparing to the control. In contrast, miR-124 inhibitor promoted macrophage cell apoptosis (Figure 7A). In addition, the role of p38 on foam cell apoptosis was also detected by flow cytometry. As shown in Figure 7B, downregulation of p38 could decrease the cell apoptosis rate while overexpression of p38 could significantly promote macrophage cell apoptosis (*P* < 0.05).

## DISCUSSION

Atherosclerosis is one of the CAD with highest mortality in western countries [23]. It is reported as active, inflammation-driven process and activation of p38 MAPK, a key regulator involved in inflammatory responses [16], in macrophages has been shown to induce the expression of pro-inflammatory cytokines in response to modified LDL incubation [24, 25]. However, few studies have conducted to investigate the upstream of p38 MAPK in the development of atherosclerosis. In this study, we predicted that miR-124 could target p38 gene by *in silico* analysis and validated their relationship by luciferase reporter assay. Next, we examined the expression of miR-124 in blood plasma and peripheral blood mononuclear cells of patients with atherosclerosis. Besides, we also tested the role of miR-124 in the animal and cell models of atherosclerosis. Our results demonstrated that miR-124 could increase the expression of anti-inflammatory cytokines, reduce the expression of pro-inflammatory cytokines, and inhibit macrophage apoptosis by targeting p38/MAPK signaling pathway in atherosclerosis pathogenesis.

As we know, atherosclerosis begins as an inflammatory response and inflammation is involved in all steps of plaque development [8]. A recent study identified miR-124 as an important modulator of cell proliferation, differentiation, and phenotype switch in human aortic vascular smooth muscle [26]. However, the role of miR-124 in atherosclerosis inflammatory response has not been systematically studied in depth. In this study, we revealed the anti-atherogenic effect of miR-124 both *in vitro* and *in vivo*. For the in vivo study, the miR-124 was delivered by using agomir with high efficiency. MiRNA agomir is a kind of miRNA agonist that has undergone special chemical modification. It mimics the endogenous miRNA to enter the miRISC complex to regulate the expression of target gene. Compared with miRNA mimics, miRNA agomir has higher stability and activity in animals, and is more likely to be enriched in target cells through the cell membrane and tissue gap [27].

The oxidation of LDL is the first event in foam cell formation [28, 29]. At present study, we treated RAW264.7 macrophage cells with ox-LDL for 48 h, and macrophages were induced into foam cells. The miR-124 level was significantly decreased in foam cells, and p38 was markedly upregulated in foam cells at both mRNA and protein levels. To verify if p38 was targeted by miR-124, we performed qRT-PCR, western blot, and luciferase report assays. Our results demonstrated that miR-124 inhibited the mRNA translation of p38 in RAW264.7 macrophage cells by targeting its 3’-UTR.

In the case of atherosclerosis, cytokines are divided into two groups: pro- and anti-atherogenic cytokine, according to their effects on the formation and progression of atherosclerotic plaque [30]. Cytokine IL-6, TNF-α, and MCP-1 promote atherosclerosis and are classified as pro- cytokines, while cytokine IL-10 and TGF-β suppress the formation and progression of atherosclerosis and are categorized as anti-atherogenic cytokines [30]. To further investigate the role of miR-124 in inflammatory responses in the process of atherosclerosis, the expression of pro- cytokine IL-6, TNF-α, and MCP-1 and anti-atherogenic cytokine IL-10 and TGF-β were evaluated in ox-LDL treated macrophage foam cells and atherosclerosis model mice. ELISA results suggested that miR-124 overexpression markedly suppressed the expression levels of IL-6, TNF-α, MCP-1 and IL-1β, while significantly promoted the expression levels of IL-10 and TGF-β. Inhibition of miR-124 reversed the result. ELISA was further performed on the blood serum samples from atherosclerosis model mice. Our results showed that agomir-124 significantly suppressed the protein levels of IL-6, TNF-α, MCP-1 and IL-1β, whereas increased the protein levels of IL-10 and TGF-β *in vivo*. These findings implied that miR-124 inhibited the expression of pro-cytokines and promoted the expression levels of anti-atherogenic cytokines in the process of atherosclerosis.

IHC assays were conducted on the thoracic aorta specimens from atherosclerosis model mice. The IHC results demonstrated that agomir-124 treatment decreased the staining area of macrophage marker CD68, pro-inflammatory cytokine IL-6, TNF-α, MCP-1, IL-1b, and pro-apoptotic factor Bax and caspase 3 comparing to the corresponding controls, while increased the staining area of anti-inflammatory cytokine IL-10 and TGF-β and anti-apoptotic factor Bcl-2 comparing to their corresponding controls, suggesting that miR-124 not only inhibited inflammatory responses, but also prevented cell apoptosis during atherosclerosis development.

Endothelial cell apoptosis is a key event in atherosclerosis and associated with atherosclerotic plaque development and thrombosis [31]. The previous study reported that the Bax and caspase 3 expressions were increased, while Bcl-2 expression was decreased in atherosclerosis plaque [32]. Consistently, our IHC assay results showed that Bax and caspase 3 was highly expressed, while Bcl-2 had a low expression in atherosclerotic lesions. The administration of agomir-124 significantly increased the expression level of Bcl-2, while decreased the levels of Bax and caspase 3 in the atherosclerotic lesions from atherosclerosis model mice. Western blot assays performed on the foam cells transfected with the miR-124 mimics, inhibitor or negative control further confirmed these results. These findings indicated that miR-124 may inhibit cell apoptosis in atherosclerosis plaque. Macrophage apoptosis is crucial to acute plaque rupture and plays opposite roles in atherosclerotic plaque development [22, 33]. In the early stage, macrophage apoptosis decreases macrophage accumulation and retards plaque progression [22]. In the late stage, macrophage apoptosis promotes the development of the necrotic core of advanced plaques, leading to plaque disruption and acute thrombosis [34]. In order to unveil the function of miR-124 on macrophage apoptosis in the progression of atherosclerosis, RAW264.7 macrophage cells were treated with ox-LDL after the transfection with miR-124 mimics, inhibitor or negative control. Annexin V/PI staining demonstrated that overexpression of miR-124 inhibited the apoptosis of macrophage foam cells, while suppression of miR-124 activated macrophage apoptosis. Our results implied that miR-124 could inhibit macrophage apoptosis in the process of atherosclerosis.

There are some inevitably limitations in this study. First, only luciferase reporter assay was performed to investigate the association between miR-124 and p38. Other experiment, such as RNA immunoprecipitation or RNA pull-down was not performed. Second, most of the *in vivo* experiments were performed on only one time point and time-course spatiotemporal regulations of the pro- and anti-atherogenic cytokines were not investigated. Third, RNA-seq or ATAC-seq was not performed to investigate the driver which changes the expression level of miR-124 and p38. Forth, further loss-of-function experiments should be performed to investigate whether miR-124 regulated expression of pro- and anti-inflammatory cytokines through regulating expression of p38 during atherosclerosis development. Therefore, further investigation of the role of miR-124 and p38 in atherosclerosis is still warranted.

**Figure 8:**
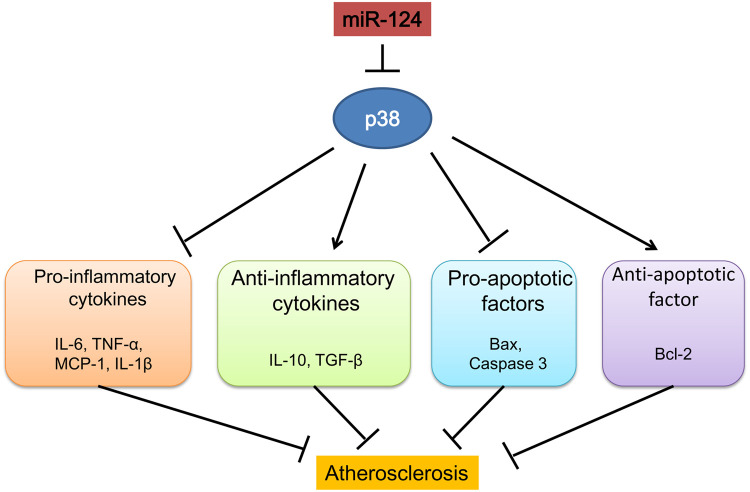
**Schematic diagram summarizing the mechanisms of how microRNA-124 play a role in atherosclerosis.**

In conclusion, our study demonstrated that miR-124 suppressed inflammatory responses and inhibited macrophage apoptosis during the development atherosclerosis by targeting p38/MAPK pathway (Figure 8). MiR-124 could be a new therapeutic target in the treatment of atherosclerosis and CAD.

## MATERIALS AND METHODS

### Patients

Eighty patients who underwent coronary angiography in our hospital for chest pain were recruited in the study. CAD was defined as ≥ 50% coronary stenosis in at least one vessel by a interventional cardiologist performing coronary angiography. The exclusion criteria included: 1) patients with history of blood diseases, liver and kidney diseases, infectious diseases or familial hypercholesterolemia; 2) patients with history of percutaneous coronary intervention, coronary artery bypass graft surgery, heart failure, or statin allergy. At last, 40 patients were recruited in CAD group and 40 patients with no obvious stenosis (normal or with < 50% coronary stenosis) under coronary angiography were assigned into control group. The baseline characteristics of all included patients were recorded. Peripheral blood was collected from patients and the monouclear cells and plasma were further isolated and stored at -80 °C for further analysis. This study obtained approval from the Ethics Committee of the Second Affiliated Hospital of Tianjin Medical University (No.KY2019K070). All the participants provided written informed consent.

### Cell culture

RAW264.7 cells and HEK293T cells were purchased from the ATCC (Manassas, VA, USA) and cultured in RPMI-1640 containing 10% fetal bovine serum (FBS), streptomycin (100 μg/ml), penicillin G (100 U/ml) and L-glutamine (2 mM) at 37°C and 5% CO_2_. Before cell transfection, RAW264.7 cells were cultured with oxidized low-density lipoprotein (ox-LDL, 50 nM) for 24 h to induce into foam cells. Foam cells were confirmed by oil red O staining according to previous study. Images were taken from at least three randomly fields using IX73 microscopy (Olympus, Japan) [35].

### Cell transfection

MiR 124 mimic (Ribobio, Guangzhou, China), miR 124 inhibitor (Ribobio), *p38* siRNA (1μg/μl, Ribobio) or their corresponding negative control (NC, Ribobio) were transfected into RAW264.7 cells with lipofectamine 2000 (Invitrogen, Carlsbad, CA, USA).

### Animal experiments

Forty male ApoE-/- mice with a C57B/L6J background, aged 6-8 weeks, weighing 20-25g, were bought from Experimental Animals Center of Peking University (Beijing, China) and were housed in animal center of Tianjin Medical University. The *ApoE*-/- mice were assigned into 4 groups randomly, with 10 mice in each group, including control group, model group, miR-124 agomir (a kind of chemically modified miRNA agonist) group and miR-124 agomir control group. The treatment procedures of each group were similar as our previous study [36]. Especially, 10 nmol miR-124 agomir or mismatched miR-124 agomir control in 0.1 ml saline buffer (Ribobio) were chemically modified and cholesterol conjugated, and were injected via tail-vein into *ApoE*-/- mice every 5 days for 4 weeks. At week 20, blood plasma from atherosclerosis model mice were prepared and glucose and lipid profile levels were measured using automated clinical chemistry analyzer. The thoracic aorta of mice was immediately harvested and stored at -80°C after being sacrificed by cervical dislocation. All animal study protocols were approved by the Animal Ethics Committee of Tianjin Medical University.

### RNA extraction and qRT-PCR

Total RNA from plasma, thoracic aorta tissues and foam cells were extracted and reverse transcribed into cDNA using miRcute miRNA First-strand cDNA Synthesis Kit (TIANGEN, Beijing, China). qPCR was performed on ABI7500 (Applied Biosystems). GAPDH and U6 served as the internal controls for mRNA and miRNA, respectively. All the PCR experiments were performed in triplicate and repeated at least 3 times. The primers in qRT-PCR were shown in Table 3.

**Table 3:** Primer sequence for qRT-PCR.

**Target**	**Direction**	**Sequence**
U6	Stem-loop RT	AACGCTTCACGAATTTGCGT
	sense	5’-CTCGCTTCGGCAGCACA-3’
	antisense	5’-AACGCTTCACGAATTTGCGT-3’
miR-124	Stem-loop RT	GTCGTATCCAGTGCAGGGTCCGAGGTATTCGCACTGGATACGACGGCATT
	sense	5’-UAAGGCACGCGGUGAAUGCC-3’
	antisense	5’-GTATCCAGTGCAGGGTCCGAGGT-3’
p38	sense	5′-CCTGATGATGAGCCTGTTGC-3′
	antisense	5′-GAGAAGGTCTTCCCCTCACA-3′
GAPDH	sense	5′-TGCACCACCAACTGCTTAG-3′
	antisense	5′-GATGCAGGGATGATGTTC-3′

### Western blot

Total proteins were extracted from thoracic aorta tissues or foam cells and western blot was performed to detect the protein expression of p38 (1:5,000), p-p38 (1:1,000), ERK (1:2,000), p-ERK (1:1,000), JNK (1:2,500), and p-JNK (1:2,000) as indicated in a previous study [36].

### Enzyme-linked immunosorbent assay (ELISA)

The amounts of cytokines in the cell supernatants of RAW264.7 cells were quantified using ELISA kits (R&D Systems, Minneapolis, MN, USA).

### Histopathologic analysis and immunohistochemistry (IHC) assay

H&E staining and IHC assay were used to evaluate the atherosclerotic lesions and protein expressions as described in reference [36, 37].

### Luciferase reporter assay

The miRNA binding sites in the 3’- untranslated region (UTR) of p38 were predicted using TargetScan 7.1 (http://www.targetscan.org/mmu_61/) and verified by luciferase reporter assay. 3’-UTR of p38, containing the predicted miR-124 target sequence, was amplified from genomic DNA of mouse bone marrow cells. The 3’-UTR mutant (GUGCCUU>CACGGAA) was generated by site directed mutagenesis with modified primers [38] (sense, p38UTR-F-X, antisense, p38UTR-R-N, p38UTR-m-F, and p38UTR-m-R). The wild-type (WT) 3’-UTR or mutated 3’-UTR was cloned into psiCHECK^TM^-2 vector (Promega, Shanghai, China) at *Xho*I and *Not*I sites, downstream of a luciferase CDS. The validity of these constructs was verified by sequencing.

RAW264.7 cells were transfected with 0.4 μg of luciferase reporter vector in the presence of 0.5 μg of miR-124 mimics, inhibitor or negative control. The luciferase activity was measured using Luciferase Reporter Assay System (Promega, Madison, WI, USA) after 48 h.

### Flow cytometric analysis

Macrophage-derived foam cells were collected in ice-cold PBS, then stained with the annexin V and propidium iodide (PI). The annexin and PI fluorescence were examined by flow cytometric analysis.

### Statistical analysis

Data were displayed as means ± standard error of the mean (SEM) and were analyzed by SPSS version 19.0 (IBM, Armonk, NY, USA). Statistical differences among groups were compared by Student’s t-test or one-way ANOVA with Bonferroni correction post-hoc analysis. *P* < 0.05 was regarded as statistically significant.

## Supplementary Material

Supplementary Figures
