# Supplementary material for: MicroRNA-124 inhibits macrophage cell apoptosis via targeting p38/MAPK signaling pathway in atherosclerosis development
Source: Aging (Albany NY). 2020 Jun 30;12(13):13005–22. doi: 10.18632/aging.103387 (PMC7377888; doi:10.18632/aging.103387)
Supplement: Supplementary Figures [file aging-12-103387-s001..pdf]

SUPPLEMENTARY FIGURES

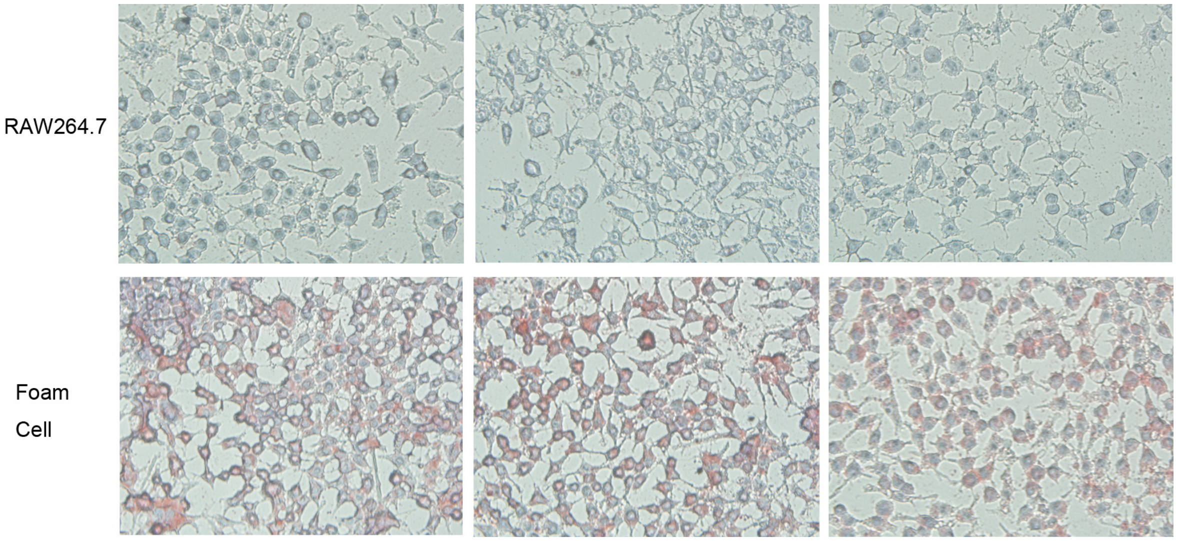

Supplementary Figure 1. Oil red O staining of Raw267 cell and foam cell.

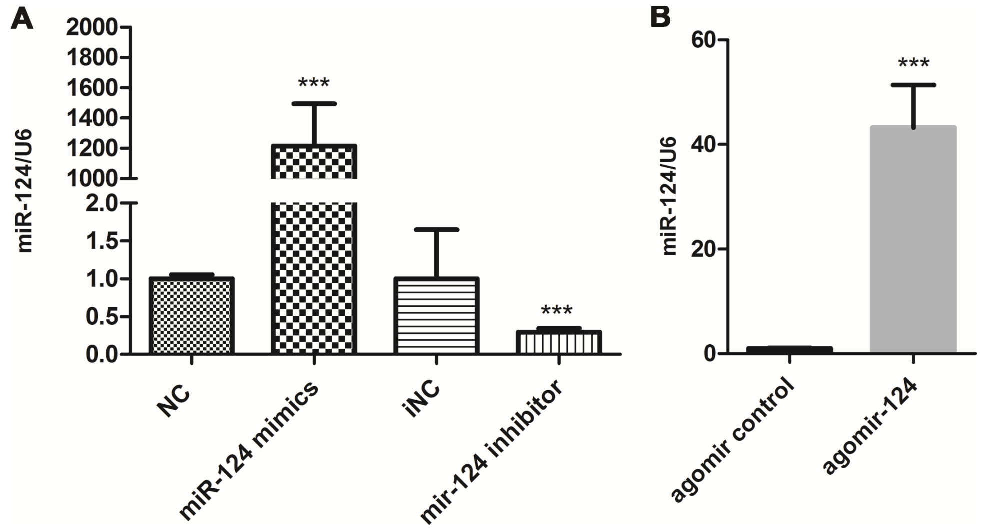

Supplementary Figure 2. Transfection rate of agomir-124 (**A**), miR-124 mimics, and miR-124 inhibitor (**B**) detected by qRT-PCR.***, $P < 0.001$ compared with control group.
